# Assisting the implementation of screening for type 1 diabetes by using artificial intelligence on publicly available data

**DOI:** 10.1007/s00125-024-06089-5

**Published:** 2024-02-14

**Authors:** Pedro F. Teixeira, Tadej Battelino, Anneli Carlsson, Soffia Gudbjörnsdottir, Ulf Hannelius, Matthias von Herrath, Mikael Knip, Olle Korsgren, Helena Elding Larsson, Anton Lindqvist, Johnny Ludvigsson, Markus Lundgren, Christoph Nowak, Paul Pettersson, Flemming Pociot, Frida Sundberg, Karin Åkesson, Åke Lernmark, Gun Forsander

**Affiliations:** 1 Diamyd Medical AB, Stockholm, Sweden; 2 University Medical Center Ljubljana, University of Ljubljana, Ljubljana, Slovenia; 3 Faculty of Medicine, University of Ljubljana, Ljubljana, Slovenia; 4 Department of Clinical Sciences, Lund University/CRC, Skåne University Hospital, Malmö, Sweden; 5 Swedish National Diabetes Register, Centre of Registers, Gothenburg, Sweden; 6 Department of Molecular and Clinical Medicine, Institute of Medicine, University of Gothenburg, Gothenburg, Sweden; 7 Global Chief Medical Office, Novo Nordisk, A/S, Søborg, Denmark; 8 Diabetes Research Institute, University of Miami, Miami, FL USA; 9 Research Program for Clinical and Molecular Metabolism, Faculty of Medicine, University of Helsinki, Helsinki, Finland; 10 Center for Child Health Research, Tampere University Hospital, Tampere, Finland; 11 Department of Immunology, Genetics and Pathology, Uppsala University, Uppsala, Sweden; 12 Department of Clinical Chemistry and Transfusion Medicine, Institute of Biomedicine, University of Gothenburg, Gothenburg, Sweden; 13 Department of Pediatrics, Skåne University Hospital, Malmö, Sweden; 14 Crown Princess Victoria Children’s Hospital and Division of Pediatrics, Department of Biomedical and Clinical Sciences, Linköping University, Linköping, Sweden; 15 Department of Clinical Sciences Malmö, Lund University, Malmö, Sweden; 16 Department of Paediatrics, Kristianstad Hospital, Kristianstad, Sweden; 17 Division of Networked and Embedded Systems, Mälardalen University, Västerås, Sweden; 18 MainlyAI AB, Stockholm, Sweden; 19 Steno Diabetes Center Copenhagen, Herlev, Denmark; 20 Department of Clinical Medicine, Faculty of Health and Medical Sciences, University of Copenhagen, Copenhagen, Denmark; 21 Department of Paediatrics, Institute for Clinical Sciences, Sahlgrenska Academy, University of Gothenburg, Gothenburg, Sweden; 22 Queen Silvia Children’s Hospital, Sahlgrenska University Hospital, Gothenburg, Sweden; 23 Department of Clinical and Experimental Medicine, Division of Pediatrics and Diabetes Research Center, Linköping University, Linköping, Sweden; 24 Department of Pediatrics, Ryhov County Hospital, Jönköping, Sweden

**Keywords:** AI, Artificial intelligence, ASSET, Children, Precision medicine, Prevention, Screening, Type 1 diabetes

## Abstract

The type 1 diabetes community is coalescing around the benefits and advantages of early screening for disease risk. To be accepted by healthcare providers, regulatory authorities and payers, screening programmes need to show that the testing variables allow accurate risk prediction and that individualised risk-informed monitoring plans are established, as well as operational feasibility, cost-effectiveness and acceptance at population level. Artificial intelligence (AI) has the potential to contribute to solving these issues, starting with the identification and stratification of at-risk individuals. ASSET (AI for Sustainable Prevention of Autoimmunity in the Society; www.asset.healthcare) is a public/private consortium that was established to contribute to research around screening for type 1 diabetes and particularly to how AI can drive the implementation of a precision medicine approach to disease prevention. ASSET will additionally focus on issues pertaining to operational implementation of screening. The authors of this article, researchers and clinicians active in the field of type 1 diabetes, met in an open forum to independently debate key issues around screening for type 1 diabetes and to advise ASSET. The potential use of AI in the analysis of longitudinal data from observational cohort studies to inform the design of improved, more individualised screening programmes was also discussed. A key issue was whether AI would allow the research community and industry to capitalise on large publicly available data repositories to design screening programmes that allow the early detection of individuals at high risk and enable clinical evaluation of preventive therapies. Overall, AI has the potential to revolutionise type 1 diabetes screening, in particular to help identify individuals who are at increased risk of disease and aid in the design of appropriate follow-up plans. We hope that this initiative will stimulate further research on this very timely topic.

**Graphical Abstract:**

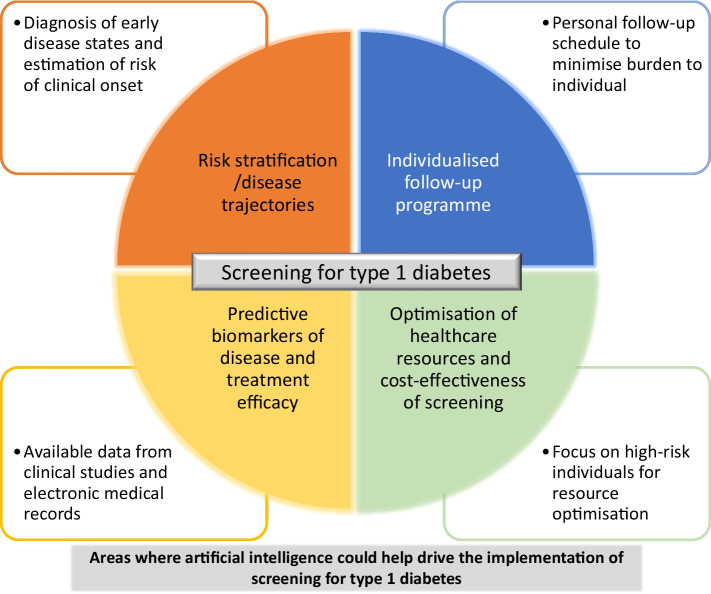

## Introduction

Type 1 diabetes is commonly regarded as an autoimmune condition that starts long before symptomatic manifestations [1]. When reduced endogenous insulin production and hyperglycaemia reach a critical threshold, individuals develop symptoms and are sometimes diagnosed in dramatic circumstances when presenting in diabetic ketoacidosis (DKA).

Insulin replacement therapy has been available to treat type 1 diabetes for 100 years, with the last 30 years witnessing the development of more efficacious insulins, more accurate insulin delivering methods and more sophisticated ways to monitor blood glucose [2]. While insulin is a life-saving treatment, it is not a cure. Therefore, every child or adult that is diagnosed with type 1 diabetes has to come to terms with a complicated and potentially dangerous treatment regimen and faces the negative long-term medical, social and economic consequences of the disease [3–5]. Even though remarkable improvements in disease management and survival have been observed during the past century, mortality rate in type 1 diabetes is still two to eight times higher than in populations without diabetes. This is reflected in a loss of life expectancy at age 20 of approximately 12 years [6].

The holy grail of type 1 diabetes clinical research is to find treatments that prevent or delay the clinical onset of disease [7]. Similar to the success of antiretroviral drugs for the treatment of HIV infection, disease-slowing treatments need to be developed for individuals with presymptomatic type 1 diabetes. A number of large observational cohort studies have followed thousands of children from birth. A wealth of information on genetics (e.g. human leukocyte antigen [HLA] genes) and biomarkers (e.g. diabetes-related autoantibodies) in type 1 diabetes, as well as on the natural history of type 1 diabetes progression, has been obtained to identify risk factors for the pathogenesis leading up to the clinical disease [8–12]. Presymptomatic screening has been advocated for genetic and serological risk variables.

Early screening allows individuals at risk and their families to prepare for a diagnosis of type 1 diabetes, although advanced knowledge of disease risk may increase the psychological burden if there is no preventive treatment or possibility of enrolling in a clinical trial [13]. Screening has also reduced the occurrence of DKA and the risk and duration of hospitalisation at diagnosis and may provide positive long-term effects on the course of the disease [14, 15]. The experience in Finland suggests that participation in prospective follow-up studies reduces the frequency of DKA in children at diagnosis of type 1 diabetes, but that genetic screening alone does not decrease DKA risk [16]. This highlights the need for predictive biomarkers and sequential follow-up of autoantibody-positive individuals. The ultimate goal is to prevent clinically overt type 1 diabetes (stage 3) by providing effective and safe treatments for individuals at high risk for the disease but still with sufficient beta cell function. These could be pharmacological interventions targeting the immune system, to replenish lost beta cells, or advanced therapies using stem cells and gene editing [13, 17, 18].

While advances in screening and prevention have been acknowledged, improvements are needed in the areas of risk prediction, operationality of screening programmes, health-economic evaluation and interactions with societal stakeholders for practical implementation.

## Using artificial intelligence to drive a precision medicine approach to type 1 diabetes

A fundamental aspect of precision medicine entails the recognition of identifiable subpopulations with variations in disease susceptibility, prognosis and treatment response. Artificial intelligence (AI) holds the promise of being a key driver of precision medicine [19] by harnessing feature information contained in available clinical datasets (see Text box 1). Through advanced algorithms, AI can uncover personalised disease trajectories and treatment responses. This is mostly achieved using machine learning (ML), a subset of AI that enables computers to learn from training datasets. Such strategies hold the potential to provide clinicians with interventions—whether they involve disease modification or prevention—tailored to the specific traits of individuals. Prediction algorithms using AI approaches for cancer [20, 21], CVD [22] and autoimmunity [23–25] have shown promising results. AI has also been applied in type 1 diabetes, for instance in optimising insulin pump settings [26], in potentially identifying predictive biomarkers [27] and for the detection of complications [28].


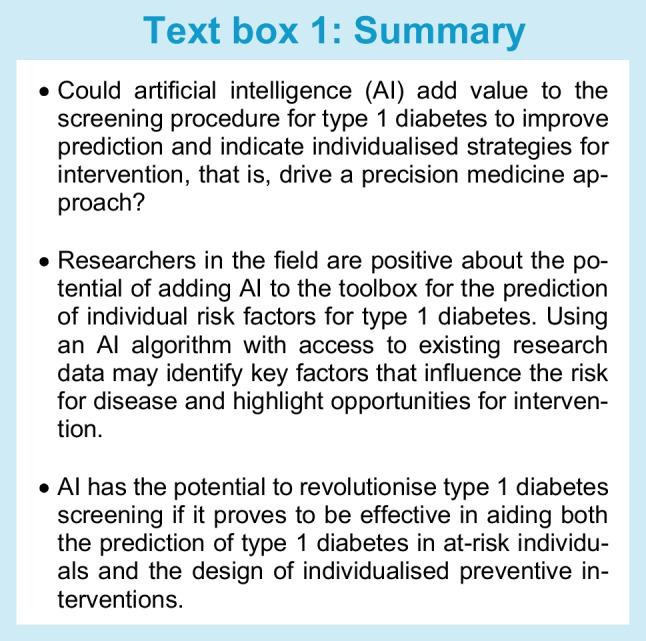


Precision prevention in type 1 diabetes is based on the ability to determine the individual risk of clinical disease onset, by using aggregated data such as genetic susceptibility, family history, environmental exposures and behavioural factors, as well as the ability to tailor personalised prevention approaches derived from such data. Seropositivity of islet autoantibodies, defined HLA haplotypes and genetic risk scores are currently the best available biomarkers for type 1 diabetes and are used to inform risk for disease development [29–31] and for the selection of individuals for clinical trials [32]. Applying precision medicine approaches that match treatments with the likely responder population is one way to account for disease heterogeneity and optimise the risk–benefit balance for the individual and society [33]. Screening for autoantibodies is often combined with genetic prescreening in cohort studies using a layered approach, with participants first undergoing genetic screening, and those with a higher genetic risk score proceeding to autoantibody testing and close follow-up. For instance, in the TEDDY (The Environmental Determinants of Diabetes in the Young) study, HLA screening at birth was an inclusion criterion [8].

AI modelling applied to disease detection has ample ability to provide an earlier diagnosis for individuals at risk [34] and to distinguish fast from slow progressors. In addition to screening for autoantibodies and HLA risk factors, analysis by AI may uncover new predictive biomarkers from variables that are already measured in clinical practice and data that are readily available in electronic medical records. Recent work has used ML of available clinical data for the early detection of individuals at high risk for pancreatic cancer [35]. These types of studies allow features that are predictive of disease risk to be identified and, in the long term, allow the design of more efficient screening strategies that take risk stratification into account.

Although there is a wealth of data available from observational studies on type 1 diabetes that have gathered invaluable clinical information over the last few decades, such datasets would still be considered ‘small data’ from an AI applicability perspective. One of the challenges of applying AI modelling to type 1 diabetes risk prediction is precisely developing strategies that do not require massive amounts of data. This approach is in line with the AI field pivoting away from the common perception that it is almost synonymous with ‘big data’ [36] to a focus on small data to deliver valuable biological insights. This could be achieved using synthetic data [37], artificially generated data that imitate the characteristics and patterns of real-world data without containing actual information from individual observations. Such data are produced using algorithms to simulate the statistical properties, distributions and relationships present in ‘authentic’ clinical datasets. Synthetic data can be used for various purposes, including testing and validating algorithms, training ML models and conducting analyses, while safeguarding the privacy and confidentiality of sensitive information present in the original data. This could be particularly helpful in tackling issues of data sharing and in balancing skewed datasets. Many cohort studies are skewed towards non-diagnosed individuals.

AI could also help to establish a second important variable of a screening programme: an individualised follow-up programme. In an ideal world, an individual seen by a general practitioner or paediatrician would benefit from type 1 diabetes risk prediction and a follow-up schedule, for example on a 0.5, 1, 5 and 10 year timescale, based on already available clinical data.

In addition to risk prediction, AI has the potential to guide pharmaceutical companies and clinical researchers with advanced effect and response prediction, to target and deliver preventive therapeutics to the individuals most likely to benefit from them [38, 39]. Such an approach also has the potential to help redesign clinical trials to approach population selection more efficiently.

## The ASSET consortium

The ASSET consortium (AI for Sustainable Prevention of Autoimmunity in the Society; www.asset.healthcare), partially funded by the Swedish Innovation Agency (VINNOVA), was established in 2021 to contribute to the type 1 diabetes research landscape. ASSET is a consortium of academic, healthcare and industry partners whose aim is to contribute a personalised prediction and prevention strategy for autoimmune diseases (see Text box 2). ASSET will investigate how AI can be applied to data in existing cohort studies to identify (1) individuals at risk for type 1 diabetes and (2) individuals who would benefit from precision secondary prevention or early intervention with therapeutic approaches. ASSET will also function as a testbed for the clinical development of prevention therapeutics, studying the articulation between screening programmes and clinical trials. Additionally, ASSET aims to analyse the organisational, economic, ethical and legal prerequisites and consequences of applying precision prevention within type 1 diabetes in the Swedish healthcare system. The aim is to proactively address obstacles that may hinder the transition of precision prevention from smaller screening initiatives to regular healthcare practice.


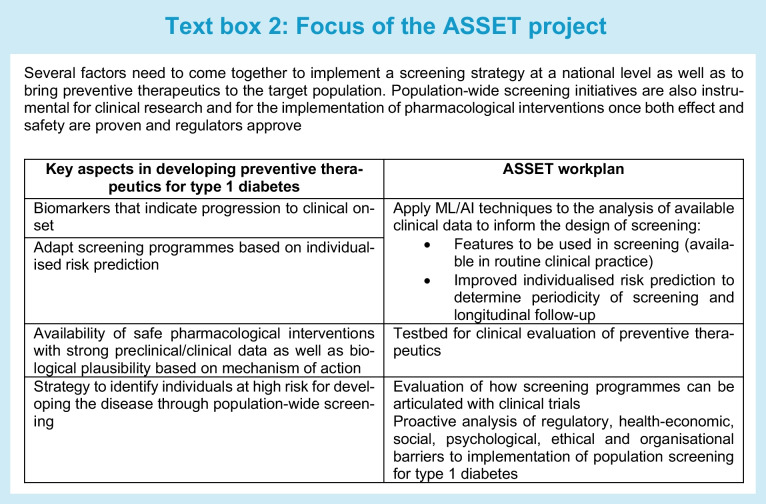


While there are other ongoing initiatives in the field that are tackling one or several of these issues, ASSET aims to approach the issues in an integrated manner, and preferably in cooperation with other ongoing programmes, but with a focus in the Nordic countries. This article describes the goals of ASSET and summarises the discussion points expressed by experts in the field who assembled at an ASSET workshop in 2022.

## Approach to ML/AI within ASSET

**Fig. 1:**
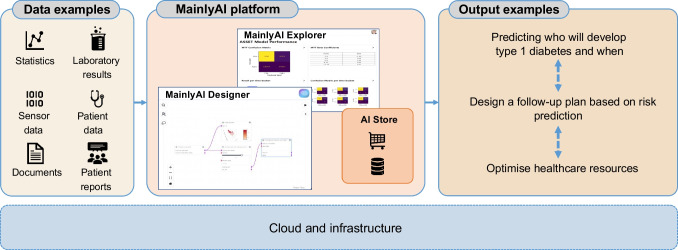
MainlyAI platform. A project can include a diversity of data types (from laboratory test results to electronic medical records, patient-reported outcomes and data from wearables). Data are pulled into the platform and then the project is designed as a flow graph in the MainlyAI designer before outputting results or integration into other systems. The figure showcases an example from ASSET; the MainlyAI Designer shows a data stream containing the TEDDY data and the MainlyAI Explorer shows an overview of model performance. The developed models allow the generation of outputs such as the prediction of type 1 diabetes. All the nodes that are developed in the project are accessible and stored in the AI Store for ease of sharing and reuse

The initial focus of ASSET is on applying ML/AI to the analysis of clinical datasets to discern patterns of disease progression and develop algorithms for individualised risk prediction. Within ASSET, the MainlyAI platform (https://mainly.ai) is used for collaboration, managing data sources and designing and performing the ML/AI studies. An overview of the MainlyAI platform is shown in Fig. 1.

The MainlyAI ML/AI tool platform has several characteristics that make it suitable for ASSET, including the following:

- Support for safe and privacy-preserving sharing: users of the tool can configure how building blocks of ML/AI projects are shared with other user groups. This means that knowledge can be shared and time can be saved when working with ML/AI projects, as already existing building blocks can easily be reused from collaborative team members, from trusted partners or from resources shared publicly on the platform. In particular, sharing of data sources and ML/AI models has proved valuable for ASSET.
- Ease of distributed collaboration: the MainlyAI platform includes a so-called platform-as-a-service in which users log in and use the tool within their web browser. This means that users can work on the same project from different geographical locations, which in turn makes it possible to invite experts from around the world to take part and contribute to project development.
- Distributed ML/AI learning approaches: techniques such as split learning or federative learning can be used in the MainlyAI platform. One advantage of such techniques is that partners can collaborate to speed up or improve the learning of a model without sharing datasets.
- Support for the full lifecycle of ML/AI projects: the tool provides support for ML/AI projects from configuration of data sources to deployment of trained models.
- Further tailoring and tool features for ASSET: as the MainlyAI platform is the property of one of the ASSET partners and under development, the tool can and will be furthered tailored towards the needs of the ASSET projects, including support for various data formats, the models and other building blocks added to the library of the tool and support for suitable data and visualisation of results.

The initial focus of ASSET is on setting up the ML pipeline (model training, testing and presentation of output) to enable the performance of several type 1 diabetes prediction studies to validate the set-up and gain insights from the datasets. The data source used to date is the TEDDY study, in which 8640 high-risk individuals have been followed from birth to age 15 years, or to a diagnosis of type 1 diabetes [40]. Current analyses are organised into two studies focusing on temporal predictive approaches. In one, we are using a multi-task temporal model to predict the risk of being diagnosed with type 1 diabetes in different time intervals. In the other, we are assessing the risk of developing type 1 diabetes and the incidence age. This will be achieved by developing two survival analysis models using Cox proportional hazards and random survival forests. Such studies represent only a first step in developing an AI model for disease prediction. Prospectively, the model(s) would need to be tested on ‘unseen’ clinical data to simulate real-world usage. The models’ predictions should then be compared with actual clinical outcomes to determine their’ accuracy, sensitivity and specificity. Collaborative efforts are needed to develop and refine AI algorithms for type 1 diabetes risk prediction and to ensure that they are based on high-quality data, as well as minimising biases, to correctly inform the design of population-wide screening programmes.

The execution of the project may be affected by the curbed availability of clinical data as well as issues around the sharing of clinical data, such as limitations of ethics permits, anonymisation and other administrative obstacles.

## Screening for type 1 diabetes in practice

Currently, screening for type 1 diabetes using a panel of islet autoantibodies is available in Sweden only through inclusion in screening studies (e.g. birth cohorts); it is not part of healthcare or standard of care for individuals prior to clinical onset. Nationwide screening initiatives, preferably integrated into the healthcare system with careful evaluation of medical, economic and psychological consequences, should be evaluated for implementation as ‘add ons’ to existing screening for other diseases in order to optimise costs and healthcare resources.

Several cohort studies screening individuals at risk for type 1 diabetes, such as TrialNet and INNODIA, have targeted relatives of those with type 1 diabetes [41]. However, a large caveat is that ∼90% of those who develop type 1 diabetes do not have a family history [42, 43] and are therefore missed in this narrow screening approach. To reach as many of those 90% as possible, general population screening for type 1 diabetes risk is unavoidable. Certain HLA haplotypes confer a high genetic risk for type 1 diabetes, and HLA screening is potentially a crucial initial step in identifying individuals who will develop autoantibodies and have a high risk of progressing to clinical type 1 diabetes [44–46]. Genetic testing to select high-risk individuals for autoantibody screening may be more cost-effective than population-wide autoantibody screening. The drawback of using genetic prescreening is that one would miss future patients carrying only a weak or limited genetic risk for type 1 diabetes. This subgroup of future patients may actually increase, as the proportion of people with high-risk HLA genotypes may decrease over time among those with newly diagnosed type 1 diabetes because of increasing environmental pressure [47].

Any screening strategy needs to consider a follow-up programme specifying a path for high-risk individuals and their families within the healthcare system. A refinement of the optimal screening strategy in terms of the variables to be tested, time points for testing and follow-up strategy is needed to inform the integration of type 1 diabetes screening into standard practice. In the long term, this is an aspect where AI may play a role through the analysis of available clinical data from cohort studies. ML/AI algorithms have the potential to integrate a multitude of variables, establishing individual risk scores to aid in stratifying populations and directing more intensive monitoring efforts towards those with elevated risk profiles. We envisage the application of AI-driven tools for optimising screening programmes as a natural evolutionary path once such programmes are established at the healthcare level. The approach would be analogous to ongoing integration of AI-based clinical support systems for breast cancer detection in ongoing screening programmes [48].

## Barriers to screening

In addition to socioeconomic considerations and issues related to public acceptance, one of the reasons often invoked for not introducing a population-wide screening programme for type 1 diabetes has been the current lack of a preventive disease-modifying treatment. The WHO recommendations based on Wilson and Jungner’s principles of screening state that it is unethical to screen for disease risk without an effective treatment [49]. The paradox is that the lack of implementation of mass screening to find at-risk individuals severely hampers the ability to develop and test preventive therapies. One key benefit of a nationwide screening programme would be the possibility to test pharmacological interventions and accelerate type 1 diabetes research. Such screening programmes would also be instrumental in the practical application of preventive therapeutics, once available. There is certainly precedent for the approval of first-in-line therapy for a disease driving implementation of screening programmes. For example, spinal muscular atrophy was added to newborn screening in the USA in 2018 after a US Food and Drug Administration (FDA)-approved therapy became available [50]. On 17 November 2022, the FDA approved teplizumab to delay the onset of stage 3 type 1 diabetes in adults and children aged 8 years and older with stage 2 type 1 diabetes [51]. This approval could be the decisive push towards the implementation of screening. More drugs are in the pipeline and societies have to be prepared to handle this breakthrough in the therapeutical arsenal.

The question of the cost-effectiveness of screening for type 1 diabetes is paramount for successful implementation in clinical practice. In addition to the costs of the actual screening process, including the costs of laboratory analysis and necessary follow-up, including management of the psychological burden for individuals and families, the costs of preventive therapies also need to be considered. Several studies have already provided cost assessments based on strategies followed in screening cohort studies, such as the Autoimmunity Screening for Kids (ASK) programme in Colorado, USA, and the Fr1da study in Bavaria, Germany [52, 53].

Targeted and efficient monitoring needs to be developed through continuous data analysis and AI-identified trends and risk patterns. By focusing monitoring efforts on high-risk individuals and efficiently allocating resources, an AI-driven programme could optimise the workflow while minimising costs, ultimately leading to a more sustainable and cost-effective approach to disease prevention and early intervention. Risk stratification and personalised monitoring programmes may be implemented by using so-called clinical decision support systems (CDSS). Application of CDSS has the potential to reduce healthcare expenditure [54], for instance by decreasing unnecessary blood testing through an optimised monitoring schedule. The potential application of CDSS to type1 diabetes screening needs to be carefully evaluated.

## Conclusion

**Fig. 2:**
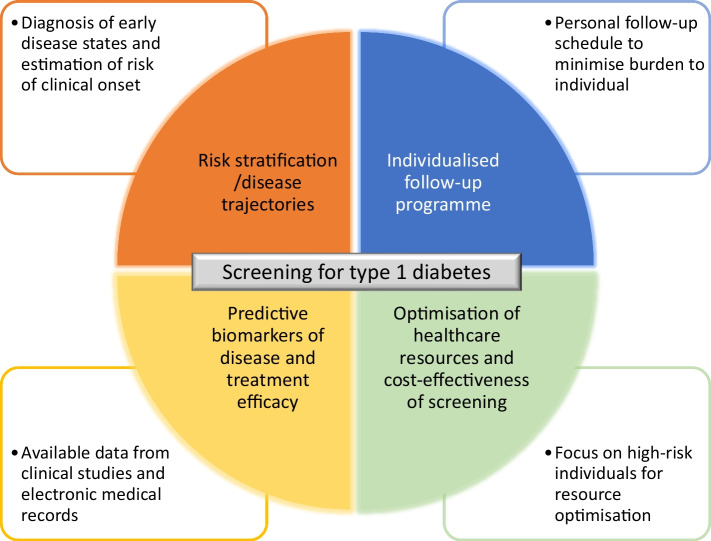
Areas where AI could help drive the implementation of screening for type-1 diabetes

Effective screening of type 1 diabetes risk in the general population would be beneficial for affected individuals and their families, academic researchers and pharma developing preventive therapeutics. Type 1 diabetes is a heterogeneous disease with distinctively progressive hallmarks that makes it amenable to a risk-based screening approach. Finding individuals at risk for the disease allows them to be monitored, to be involved in clinical trials of preventive therapeutics and, in the worst case, to be prepared for a diagnosis, avoiding an acute clinical presentation. Once treatments are available, screening will allow healthcare providers to select individuals who would benefit from a specific intervention. The design of screening programmes, including variables to screen for, how often to screen and how long to screen for, are questions that can be addressed using AI (Fig. 2). The application of AI to develop screening programmes, from identifying biomarkers that predict disease trajectory to identifying the appropriate timing of monitoring and determining cost-effectiveness, is still in its infancy. Current examples of AI application in diabetes include screening for diabetes complications and predicting hospitalisation for DKA [55, 56]. The potential benefits of AI should be weighed against the drawbacks, such as issues around data sharing, ethics and possible biases, before any AI-driven clinical support tools are introduced in population screening programmes.

The ASSET initiative is taking a broad approach, focusing on AI to help inform screening programmes, testing preventive therapeutics in a clinical setting, and evaluating the ‘implementability’ of such practices in healthcare systems. ASSET provides the means to link experts in clinical type 1 diabetes research with industry, ethics boards and public healthcare to jointly capitalise on publicly available databases for designing screening programmes, identifying individuals at risk and assisting with the use of precision medicine in personalised clinical prevention trials.

## Abbreviations

AI: Artificial intelligence
ASSET: AI for Sustainable Prevention of Autoimmunity in the Society
DKA: Diabetic ketoacidosis
HLA: Human leukocyte antigen
ML: Machine learning
TEDDY: The Environmental Determinants of Diabetes in the Young
